# Undiagnosed diabetic retinopathy in Northeast China: prevalence and determinants

**DOI:** 10.3389/fendo.2023.1263508

**Published:** 2023-11-29

**Authors:** Bo Zang, Shisong Rong, Dong Li, Xiaoxia Ding, Dongxiao Zang, Fenghua Wang, Yuanbo Liang, Gang Zhai, Kemi Feng, Zixi Zhou, Yu Wang

**Affiliations:** ^1^ Department of Ophthalmology, Fushun Eye Hospital, Fushun, Liaoning, China; ^2^ Mass Eye and Ear, Mass General Brigham, Harvard Medical School, Boston, MA, United States; ^3^ Department of Ophthalmology, Beijing Tongren Eye Center, Beijing Tongren Hospital, Capital Medical University, Beijing Ophthalmology and Visual Science Key Laboratory, Beijing, China; ^4^ Eye Hospital, Wenzhou Medical University, Wenzhou, China

**Keywords:** diabetic retinopathy, epidemiological investigation, retinal screening, public health, vision

## Abstract

**Objective:**

To report the prevalence and contributing factors of undiagnosed diabetic retinopathy (DR) in a population from Northeastern China.

**Subjects/Methods:**

A total of 800 subjects from the Fushun Diabetic Retinopathy Cohort Study were enrolled. A questionnaire assessing incentives and barriers to diagnosis of DR was administered. Logistic regression was used to identify clinical and sociodemographic factors associated with undiagnosed DR. In a prespecified subgroup analysis, we divided patients into vision-threatening diabetic retinopathy (VTDR) and non-VTDR (NVTDR) subgroups.

**Results:**

Among 800 participants with DR, 712 (89.0%) were undiagnosed. Among 601 with NVTDR, 566 (94.2%) were undiagnosed. Among 199 with VTDR, 146 (73.4%) were undiagnosed. The risk factors affecting the timely diagnosis of NVTDR and VTDR exhibit significant disparities. In multivariate models, factors associated with undiagnosed VTDR were age over 60 years (*OR* = 2.966; 95% *CI* = 1.205-7.299; *P* = 0.018), duration of diabetes over 10 years (*OR* = 0.299; 95% *CI* = 0.118-0753; *P* = 0.010), visual impairment or blindness (*OR* = 0.310; 95% *CI* = 0.117-0.820; *P* = 0.018), receiving a reminder to schedule an eye examination (*OR* = 0.380; 95% *CI* = 0.163-0.883; *P* = 0.025), and the belief that “people with diabetes are unlikely to develop an eye disease” (*OR* = 4.691; 95% *CI* = 1.116-19.724; *P* = 0.035). However, none of the factors were associated with undiagnosed NVTDR (all *P* ≥ 0.145).

**Conclusion:**

Our research has uncovered a disconcerting trend of underdiagnosis in cases of DR within our population. Addressing determinants of undiagnosed DR may facilitate early detection.

## 1 Introduction

Diabetic retinopathy (DR) stands as a predominant etiology of visual impairment among adults within the working age population who are afflicted with diabetes (1). Timely detection and judicious therapeutic interventions, such as pan-retinal photocoagulation (PRP) and anti-vascular endothelial growth factor (VEGF) injections, have been demonstrated to mitigate the risk of vision loss attributable to diabetes by 50% to 70% (2–4). However, the insidious nature of undiagnosed DR poses a significant clinical challenge, exacerbating the risk of adverse outcomes and complicating long-term management (5–7).

The efficacy of DR diagnosis is intrinsically linked to adherence to regular ophthalmic screenings. For individuals diagnosed with Type 2 Diabetes Mellitus (T2DM), immediate DR screening is imperative due to the likelihood of a pre-existing condition. Follow-up screenings are recommended annually for those with no detectable retinopathy, semi-annually to annually for mild to moderate non-proliferative diabetic retinopathy (NPDR), and quarterly for severe NPDR or proliferative diabetic retinopathy (PDR), with the latter necessitating potential expedited specialist consultation (8). In China, the burgeoning diabetes epidemic has catalyzed the initiation of regional DR screening initiatives, often orchestrated through synergistic collaborations among hospitals, community healthcare centers, and governmental agencies (9). These programs employ a spectrum of diagnostic approaches, from conventional clinical assessments to cutting-edge telemedicine platforms. Despite these efforts, the diagnostic yield remains suboptimal due to factors such as patient unawareness (10, 11), subpar screening protocols (12), limited access to healthcare resources (13), and socioeconomic and demographic disparities (13, 14).

In real-world clinical scenarios, the rate of early DR detection is disconcertingly low; an estimated 75% of DR cases in developed countries go undiagnosed (5, 15). The economic ramifications of this diagnostic gap are substantial, extending beyond the direct healthcare costs of managing advanced DR and its sequelae to include indirect costs related to productivity loss and compromised quality of life. Moreover, there is a conspicuous dearth of population-based epidemiological data on undiagnosed DR from developing nations. Hence, this study aims to elucidate the demographic, clinical, and behavioral determinants of undiagnosed DR in Northeast China, thereby offering actionable insights for enhancing the region’s DR diagnostic and management strategies.

## 2 Materials and methods

### 2.1 Participants

This study was conducted as part of the Fushun Diabetic Retinopathy Cohort Study (FS-DIRECT) from July 2012 to May 2013. Detailed information on the study design, methodology, and baseline results can be found in previous publications (16). Succinctly, the FS-DIRECT included individuals with T2DM residing in the communities of Jiangjun Street, Fushun, Liaoning Province, China. Rigorous clinical diabetes mellitus evaluations of participants were meticulously sourced from the community health center prior to the study’s initiation. Prospective candidates meeting the stipulated criteria were formally solicited for participation. Every individual diagnosed with DR from the FS-DIRECT was incorporated into our research. All participants provided signed consent forms, and the study was approved by the Institutional Review Board of Fushun Eye Hospital, adhering to the principles of the Declaration of Helsinki.

### 2.2 Socio-demographic and clinical data

All participants underwent clinical eye examinations, which involved assessments of presenting visual acuity (PVA), intraocular pressure (IOP), slit lamp examination, and fundus photography. Color fundus photography was performed on all subjects diagnosed with diabetes using a 45° nonmydriatic retinal camera (Kowa, VK-2, Tokyo, Japan). A stereoscopic macula image of each eye was captured by certified photographers after pupil dilation. The six fields of fundus photos were taken and defined as follows: Field 1 - center of the optic disc, Field 2 - center of the macula, Field 3 - temporal to the macula, Field 4 - temporal superior, Field 5 - temporal inferior, Field 6 - nasal to the optic disc (16). Individuals without DR, with ungradable retinal photographs, or with uncompleted questionnaires were excluded from the analysis.

### 2.3 Questionnaire

Interviews were conducted face-to-face in Chinese with all enrolled patients with T2DM to collect comprehensive socio-demographic data and medical conditions (16). A brief questionnaire, based on previous surveys (12, 17, 18), was utilized to gather information on the incentives and barriers by participants in attending regular retinopathy screenings conducted by certified ophthalmologists (**Supplementary Table 1**).

### 2.4 Assessment of DM

In accordance with the guidelines established by the American Diabetes Association (19), diabetes mellitus (DM) was diagnosed under the following criteria: a fasting plasma glucose (FPG) level of 7.0 mmol/L or greater; a value equal to or surpassing 11.1 mmol/L in the 2-hour oral glucose tolerance test (2-h OGTT); or the self-disclosure of prescribed diabetes medication utilization by participants.

### 2.5 Assessment of DR

We differentiate between two pivotal aspects concerning the diagnosis of DR: the objective clinical diagnosis and the subjective patient’s awareness of their DR status.

The clinical diagnosis of DR was conducted via 6-field fundus photography during the enrolment phase. Photographs were independently reviewed in detail by two graders. In cases where there was a discrepancy in the assigned levels for each eye, a consensus was reached with a third grader. The grading protocols for DR were based on the Early Treatment Diabetic Retinopathy Study (ETDRS) adaptation of the modified Airlie House classification of DR (20). The following criteria were used for grading the eyes: mild to moderate non-proliferative DR (NPDR) was characterized as levels 31-47; severe NPDR (levels 53) and proliferative DR (PDR) encompassed levels 60-85. Vision-threatening diabetic retinopathy (VTDR) was defined as the presence of severe NPDR, PDR, or clinically significant macular edema (CSME), according to the definition by the Eye Diseases Prevalence Research Group (21). Non-VTDR (NVTDR) was defined as mild or moderate NPDR or diabetic macular edema (DME) that did not meet the threshold for CSME. CSME was defined according to the ETDRS definition as thickening of the retina at or within 500 µm of the center of the macula, hard exudate at or within 500 µm of the center of the macula if associated with adjacent retinal thickening, or a zone or zones of retinal thickening of at least 1 disc area that is located at least 1 disc diameter from the center of the macula (18).

### 2.6 Patient awareness of having DR

To clarify the participants’ self-awareness of their condition, we categorized DR patients into two groups: 1) Diagnosed DR: Participants cognizant of their DR diagnosis; and 2) Undiagnosed DR: Participants oblivious to their DR condition (**Supplementary Table 2**).

The participants were asked the following two questions:

1. Have they ever received a diagnosis of DR or been informed by a doctor about eye diseases or eye problems related to their diabetes?
2. Have they ever undergone laser treatment for their diabetic eye disease?

Participants were considered undiagnosed for DR if they did not answer ‘yes’ to both questions. Participants with VTDR, were considered undiagnosed if they answered “no” to the first question and had no laser scars visible in retinal photography.

### 2.7 Assessment of visual acuity

PVA was measured for each participant using their current correction, such as glasses or contact lenses, at the time of the examination. The measurement was performed following the protocol of the ETDRS, using the logMAR visual acuity chart (Precision Vision, USA) at a distance of 4 meters for both eyes. The modified World Health Organization (WHO) definition of visual impairment (VI) was used, with LogMAR > 0.48 (20/60) to ≤1.30 (20/400) indicating VI, and LogMAR > 1.30 (20/400) indicating blindness (22). Unilateral VI or blind was categorized using the worse-seeing eye.

### 2.8 Statistical analyses

The baseline characteristics of participants in the FS-DIRECT were summarized using proportions to represent categorical factors. Chi-square tests were utilized to compare the characteristics of participants across different DR diagnosis statuses. Pairwise comparisons among multiple groups were conducted using Bonferroni correction.

Logistic regression models were then constructed to assess the associations between different classifications of PVA, specifically pertaining to the better-seeing eye and the worse-seeing eye, and the diagnosis status of DR. These associations were examined in both crude models and models adjusted for age, gender, and duration of diabetes. To determine the statistical significance of the variations in odds ratio estimates between the two PVA exposures and diagnosed DR, a cluster sandwich estimator was utilized.

To identify which items are associated with undiagnosed DR, we conducted a series of analyses. Firstly, we employed univariate analyses to explore the relationships between a range of demographic variables, clinical characteristics, barriers to attendance, and diagnosed DR. We then incorporated items demonstrating significant univariate associations into a multivariate logistic regression model. These analyses were performed for the overall population, as well as for two specific subgroups: non-VTDR (NVTDR) and VTDR. We also investigated whether there was a multiplicative interaction between each item correlated with the diagnosis status of NVTDR/VTDR and the predetermined variables such as age, sex, education level, income, occupation, duration of diabetes, and HBA1c levels. Analyses within subgroups defined by these variables were performed if the *P* value for interaction was <0.05. All statistical analyses were conducted using R software (version 4.0.4). A two-sided *P*-value of less than 0.05 was considered statistically significant.

## 3 Results

### 3.1 Patients demographics and diagnosis status by DR severity

This study included a total of 800 participants, of which 88 were diagnosed with DR and 712 were undiagnosed (**Tables 1**, **2**). When stratified by DR severity, 53 (26.6%) of those with VTDR were previously diagnosed, whereas 38 (28.8%) of those with CSME and 43 (55.8%) of individuals with PDR were previously diagnosed. Notably, only 55 (5.8%) of those with NVTDR and 5 (6.5%) of those with severe NPDR had a prior DR diagnosis (**Table 1**).

**Table 1:** Diagnosis status of different severity of diabetic retinopathy.

	N	Diagnosed	Undiagnosed
ALL DR	800	88.0 (11.0)	712.0 (89.0)
NVTDR	601	55.0 (5.8)	566.0 (94.2)
VTDR	199	53.0 (26.6)	146.0 (73.4)
CSME	132	38.0 (28.8)	94.0 (71.2)
Severe NPDR	77	5.0 (6.5)	72.0 (93.5)
PDR	77	43.0 (55.8)	34.0 (44.2)

Data are presented as frequency (%).

DR, diabetic retinopathy; NVTDR, non-vision-threatening diabetic retinopathy diabetic retinopathy; VTDR, vision-threatening diabetic retinopathy; CSME, clinically significant macular edema; NPDR, non-proliferative diabetic retinopathy; PDR, proliferative diabetic retinopathy.

**Table 2:** Baseline characteristics of participants.

	Total	Diagnosed DR	Undiagnosed DR	*P* Value^*^
	(N = 800)	(N = 88)	(N = 712)	
Age				0.415
<50	97 (12.1)	16 (18.2)	81 (11.4)	
50-60	279 (34.9)	31 (35.2)	248 (34.8)	
60-70	298 (37.3)	29 (33.0)	269 (37.8)	
≥70	126 (15.8)	12 (13.6)	114 (16.0)	
Sex				0.413
male	314 (39.3)	31 (35.2)	283 (39.7)	
female	486 (60.8)	57 (64.8)	429 (60.3)	
Ethnicity				0.930
Han	751 (94.1)	83 (94.3)	668 (94.1)	
Other	47 (5.9)	5 (5.7)	42 (5.9)	
Occupation				0.335
Employed	90 (11.3)	14 (15.9)	76 (10.7)	
Unemployed	72 (9.0)	8 (9.1)	64 (9.0)	
Retired	638 (79.8)	66 (75.0)	572 (80.3)	
Religion				0.966
No	698 (87.4)	77 (87.5)	621 (87.3)	
Yes	101 (12.6)	11 (12.5)	90 (12.7)	
Education				0.568
Illiteracy or primary school	518 (64.8)	54 (62.1)	464 (65.2)	
High school or above	281 (35.2)	33 (37.9)	248 (34.8)	
Marital status				0.258
Without a partner	134 (16.8)	11 (12.5)	123 (17.3)	
With a partner	666 (83.3)	77 (87.5)	589 (82.7)	
Income (Yuan/Month)				0.870
<3000	327 (41.2)	37 (42.0)	290 (41.1)	
≥3000	466 (58.8)	51 (58.0)	415 (58.9)	
Duration of diabetes (years)				<0.001
<5	205 (25.6)	14 (15.9)	191 (26.8)	
6-10	254 (31.8)	18 (20.5)	236 (33.1)	
11-15	184 (23.0)	23 (26.1)	161 (22.6)	
≥15	157 (19.6)	33 (37.5)	124 (17.4)	
Mean HbA1c (%)				0.960
<7.0	241 (30.3)	27 (31.0)	214 (30.2)	
7.0-9.0	288 (36.2)	32 (36.8)	256 (36.1)	
≥9.0	267 (33.5)	28 (32.2)	239 (33.7)	
LDL (mmol/L)				0.102
<2.6	188 (23.4)	26 (29.9)	154 (21.7)	
≥2.6	616 (76.6)	61 (70.1)	555 (78.3)	
TC (mmol/L)				0.562
<5.18	314 (39.4)	37 (42.5)	277 (39.1)	
≥5.18	482 (60.6)	50 (57.5)	432 (60.9)	
Unilateral Visual status				<0.001
No VI	538 (67.3)	40 (45.5)	498 (69.9)	
VI	192 (24.0)	31 (35.2)	161 (22.6)	
Blind	70 (8.8)	17 (19.3)	53 (7.4)	
Bilateral Visual status				<0.001
No VI	538 (67.3)	40 (45.5)	498 (69.9)	
Unilateral VI or blind	139 (17.4)	22 (25.0)	117 (16.4)	
Bilateral VI or blind	123 (15.4)	26 (29.5)	97 (13.6)	

Data are presented as frequency (%).

^*^P value with Chi-square tests.

DR, diabetic retinopathy; HbA1c, glycosylated haemoglobin A1c; LDL, low-density lipoprotein; TC, total cholesterol; VI, visual impairment.

### 3.2 Demographic factors and DR diagnosis

The majority of participants in both the diagnosed DR and undiagnosed DR groups were of Han Chinese ethnicity, had no religious affiliation, and were retired (**Table 2**). There were no statistically significant differences between diagnosed and undiagnosed DR groups in terms of age (*P* = 0.415), sex (*P* = 0.413), ethnicity (*P* = 0.930), religion (*P* = 0.966), education (*P* = 0.568), marital status (*P* = 0.258), income (*P* = 0.870), glycosylated haemoglobin A1c (*P* = 0.960), low-density lipoprotein (*P* = 0.102) and total cholesterol (*P* = 0.562).

### 3.3 Duration of diabetes and DR diagnosis

In subsequent analysis, statistically significant differences were observed among the groups in terms of duration of diabetes (*P* < 0.001) and PVA (*P* < 0.001). Participants with a duration of DR for ≥ 15 years had a significantly higher proportion of diagnosed cases (21.0%) compared to those with a diagnosis of DR for < 5 years (6.8%) and those with DR for 6-10 years (7.1%).

### 3.4 Visual Impairment and DR Diagnosis

Based on unilateral classifications of PVA, participants without VI had a lower proportion of diagnosed DR (7.4%) compared to those with VI (16.1%, *P* = 0.002) or blindness (24.3%, *P* < 0.001). Upon employing bilateral classifications, no discernible difference was observed in the proportion of individuals diagnosed with DR between patients experiencing unilateral VI or blindness and those with bilateral VI or blindness. **Table 3** demonstrated that VI or blindness, when based on the better-seeing eye, was associated with a reduced prevalence of undiagnosed DR (*OR* = 0.376, 95%*CI*: 0.227, 0.624), and an even more attenuated proportion when estimated upon the worse-seeing eye (*OR* = 0.358, 95%*CI*: 0.229, 0.561). Moreover, the difference between the ORs calculated from worse-seeing or better-seeing eyes was not statistically significant (*P* = 0.978) (**Table 3**). And these associations remained unaltered after adjusting for age, sex, and duration of diabetes.

**Table 3:** Associations between different classification of visual acuity and undiagnosed diabetic retinopathy.

	VA classification	*OR* (95%*CI*)	*P* value^‡^
Model 1^*^			0.960
	Worse eye VI or blind	0.358 (0.229, 0.561)	
	Better eye VI or blind	0.376 (0.227, 0.624)	
Model 2^†^			0.978
	Worse eye VI or blind	0.380 (0.239, 0.604)	
	Better eye VI or blind	0.417 (0.248, 0.703)	

^*^Crude regression model without any correction.

^†^Age, sex, and duration of diabetes adjusted.

^‡^Odd ratio estimate comparison using cluster sandwich estimator for estimating cross-model covariance matrices.

VA, visual acuity; VI, visual impairment; OR, odd ratio.

### 3.5 Factors associated with undiagnosed DR

**Figure 1:**
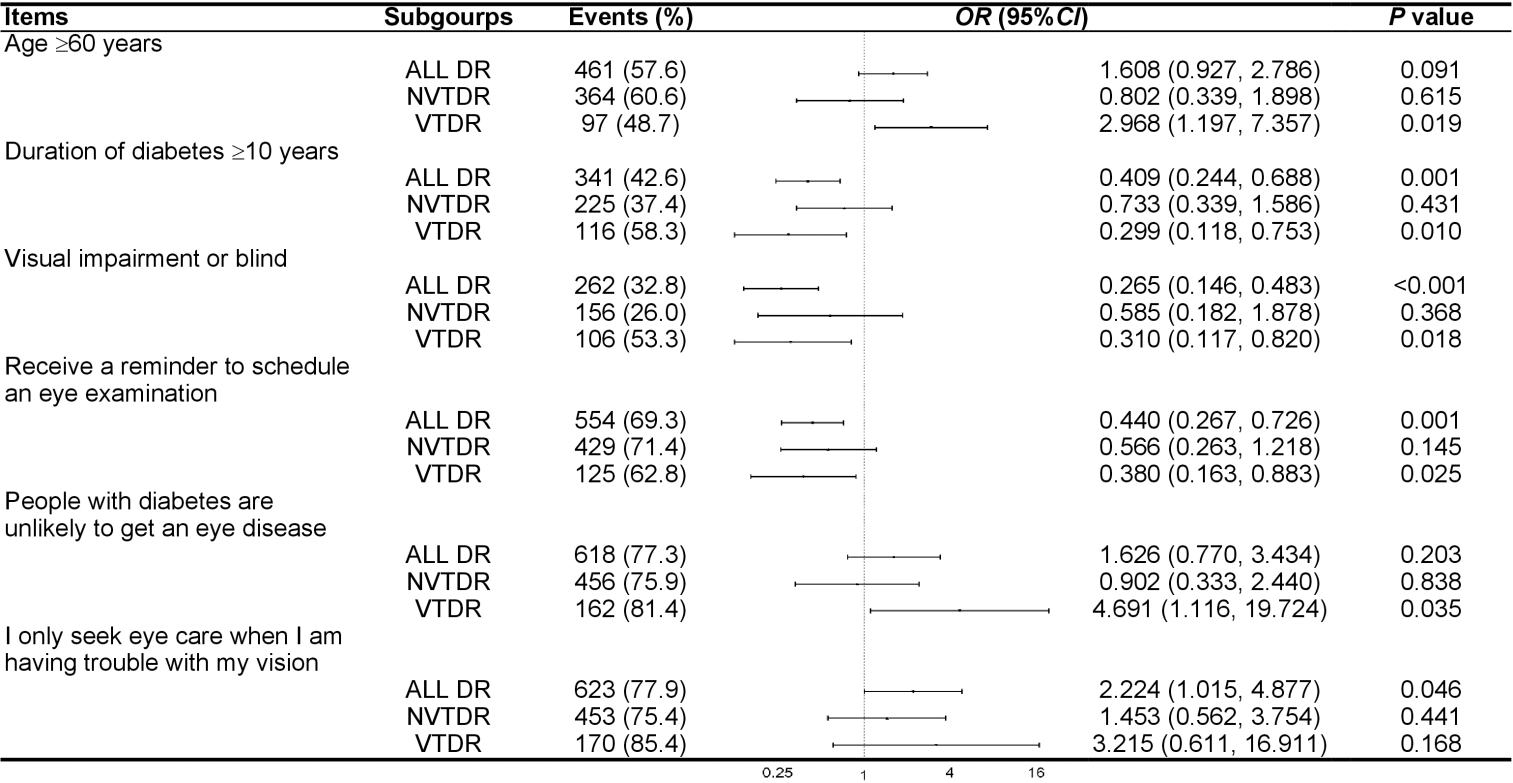
Subgroup analysis of factors associated with undiagnosed diabetic retinopathy. OR, odd ratio; CI, confidence interval; DR, diabetic retinopathy; NVTDR, non-vision-threatening diabetic retinopathy; VTDR, vision-threatening diabetic retinopathy.

Items that were significantly associated with undiagnosed DR were analyzed in all DR patients and several subgroups. **Figure 1** presented the items related to undiagnosed DR, stratified by the severity of DR. Among all DR patients, individuals who only sought eye care when experiencing vision problems were associated with a higher prevalence of undiagnosed DR (*OR* = 2.224; 95% *CI* = 1.015-4.877; *P* = 0.046). Conversely, patients with a diabetes duration of over 10 years (*OR* = 0.409; 95% *CI* = 0.244-0.688; *P* = 0.001), VI or blindness in the worse eye (*OR* = 0.265; 95% *CI* = 0.146-0.483; *P* < 0.001), and those who received a reminder to schedule an eye examination (*OR* = 0.440; 95% *CI* = 0.267-0.726; *P* = 0.001) were associated with a lower likelihood of undiagnosed DR. In the subgroup with VTDR, these relationships were attenuated but remained statistically significant for the duration of diabetes over 10 years (*OR* = 0.299; 95% *CI* = 0.118-0753; *P* = 0.010), VI or blindness in the worse eye (*OR* = 0.310; 95% *CI* = 0.117-0.820; *P* = 0.018), and receive a reminder to schedule an eye examination (*OR* = 0.380; 95% *CI* = 0.163-0.883; *P* = 0.025). Age over 60 years (*OR* = 2.968; 95% *CI* = 1.197-7.357; *P* = 0.019) and the belief that “people with diabetes are unlikely to develop an eye disease” (*OR* = 4.691; 95% *CI* = 1.116-19.724; *P* = 0.035) were associated with a reduced probability of receiving a diagnosis in patients with VTDR. In the subgroup with NVTDR, none of the factors were associated with undiagnosed NVTDR.

### 3.6 Interaction effects

**Figure 2:**
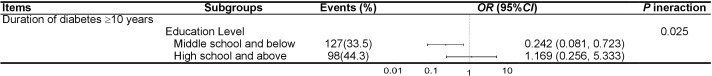
Subgroup analysis of factors associated with undiagnosed non-vision-threatening diabetic retinopathy. OR, odd ratio; CI, confidence interval.

We did not observe any clinical characteristics that modified the associations between risk factors and undiagnosed VTDR. However, we did identify a significant interaction between education level and duration of diabetes (*P* interaction = 0.025), with the inverse associations with undiagnosed NVTDR being stronger in patients with a middle school education or below (*OR* = 0.242; 95% *CI* = 0.081-0.723; *P* = 0.011; **Figure 2**).

## 4 Discussion

Our community-based study provides the prevalence and contributing factors of undiagnosed DR in Northeast China. Our study revealed that 89.0% of DR patients remained undiagnosed. This finding aligns with the Singapore Epidemiology of Eye Diseases Survey (SEED) (83.3%) (5) and the National Health and Nutrition Examination Survey (NHANES) in the USA (70.1%) (23). Furthermore, we identified an even higher proportion of undiagnosed cases among patients with NVTDR (93.5%). 71.2% of patients with VTDR were unaware of their condition, which significantly exceeds the 27.3% of undiagnosed VTDR cases reported by the SEED (5) and the 19% reported by the Diabetic Retinopathy Inpatient Study (DRIPS) (6). These findings highlight the inadequate screening efforts for DR in our population.

Our research sought to uncover the reasons behind the high rates of undiagnosed DR. We found that patients without VI were more likely to be undiagnosed, which is consistent with the findings of the SEED (5). The asymptomatic nature of DR often results in individuals presenting with advanced stages of the disease when they first consult an ophthalmologist, which is a concerning trend (15, 24–26). Another noteworthy finding is that subjective visual disturbance was not included in the final model, indicating that objective visual impairment is more of a predictor of diagnosed DR than subjective visual function. This could be due to the fact that in the early stage of DR, patients may already experience changes in contrast sensitivity (27), color discrimination (28), and low luminance VA (29). However, these symptoms may not prompt patients to seek medical care until their central vision is affected. Therefore, these findings emphasize the importance of educating individuals about diabetes and its potential eye complications, regardless of the exemption from VI. One reassuring finding is that we observed no difference in the proportion of undiagnosed cases between patients with bilateral VI and those with unilateral VI. Additionally, regression analysis showed undiagnosed cases were more strongly associated with the worse-seeing eye than the better-seeing eye. This suggests that patients tend to seek medical attention when one eye experiences VI rather than waiting until both eyes are affected.

In terms of demographic variables, our results were coupled with the previous research (5, 30), indicating that a longer duration of T2DM is associated with a lower proportion of undiagnosed DR. Over time, patients with diabetes are more likely to receive regular medical supervision, education about potential complications, and understand the importance of regular check-ups, including eye examinations. Consequently, they may be more proactive in monitoring their health, leading to earlier detection of DR.

Moreover, we found that advanced age was associated with a higher proportion of undiagnosed VTDR. As individuals age, they may experience other co-existing age-related conditions, and other health conditions take priority, resulting in delayed diagnosis of DR.

Although it is known that individuals living with close family members, particularly a partner, tend to exhibit better attendance at medical examinations (31), our study failed to find a significant association in this regard. Additionally, in line with earlier studies, we did not observe any associations between undiagnosed DR and factors such as gender (24, 32), ethnicity (24), or marital status (32).

Our study shed light on patient-reported incentives and barriers for undiagnosed DR. Since our study initiated the attempt to address this issue, direct comparisons with other studies were not possible. However, there is circumstantial evidence from studies examining the reasons for low adherence to screening guidelines that support our findings (12, 18, 33, 34).

Our study reveals that a considerable proportion (69.3%) of patients with DR reported not receiving information from their internists regarding the need for an eye examination. Additionally, we found 77.9% of participants stated that they only felt the need to seek such examinations when they experienced problems with their eyesight. Furthermore, only 22.3% of participants had visited an ophthalmologist. An alarming 75.1% of T2DM patients expressed unawareness of the recommended frequency of eye checks. In contrast, a study conducted in Saudi Arabia revealed that 73.3% of diabetic patients were aware of the need for regular eye check-ups (35). The significant knowledge gap observed in our study regarding screening for DR may be attributed to a lack of understanding about the importance of eye screening. These findings highlight the critical importance of effective inter-specialty communication in the comprehensive management of complex chronic diseases like diabetes. Healthcare providers, particularly general practices and endocrinologists, should adopt a proactive role in disseminating this crucial information, including the necessity and frequency of diabetic eye screening, addressing misconceptions, and promoting regular ophthalmological examinations (36).

The fact that over 77% of patients demonstrated awareness of the potential link between DM and DR is noteworthy. However, it is concerning that only 28.5% of patients are aware that DR could lead to blindness, which aligns with previous studies (37). These findings emphasize the necessity for targeted patient education initiatives that focus on raising awareness about the risks and potential consequences of DR, specifically highlighting the risk of blindness. Such a proactive approach has the potential to facilitate earlier detection of DR, enhance patient outcomes, and ultimately reduce vision loss associated with diabetes (2–4).

As the duration of diabetes increases, individuals with lower levels of education are more likely to have NVTDR, while this association is not observed among individuals with higher levels of education. These findings imply the existence of potential communication gaps between these patients and healthcare providers. These patients may have difficulty fully comprehending the health information provided, leading to challenges in making informed decisions about their healthcare. This, in turn, increases the risk of undiagnosed NVTDR. Additionally, despite the similar proportion of patients with NVTDR and VTDR attending regular screenings (21.0% vs. 19.1%), we observed a significantly lower diagnosis rate of DR in the former group (5.5% vs. 26.6%). It is important to note that the research was conducted in a third-tier city, which typically has limited healthcare infrastructure and a scarcity of ophthalmologists specialized in retinal compared to larger urban centers. Even if patients undergo regular follow-up visits, the mild manifestations of NVTDR pose challenges in achieving accurate diagnoses (36). This could be a major contributing factor to the underdiagnosis of NVTDR in our population.

While traditional methods such as direct and indirect ophthalmoscopy have served as foundational tools in DR screening, their limitations in sensitivity and specificity, especially in non-dilated pupils, are evident. The advent of advanced imaging techniques, such as Fluorescein Angiography (FA) and Optical Coherence Tomography (OCT), has revolutionized the precision with which DR can be detected and monitored. With the rapid advancements in technology, the future holds promise for even more efficient and accessible DR screening methods. Artificial intelligence and machine learning algorithms, for instance, are being integrated into retinal imaging tools to provide instant, automated assessments of DR severity (38). Such innovations could significantly reduce the time and expertise required for DR screenings, facilitate the early diagnosis of NVTDR, making it more widely available and potentially more cost-effective.

The strengths of our study lie in the inclusion of a large community-based sample, the utilization of six-field retinal photographs for assessing DR, and the adoption of masked grading to ensure accurate evaluation. In order to obtain a precise and comprehensive understanding, it is imperative to delineate the limitations inherent to this study. Primarily, the data utilized is circumscribed to the FS-DIRECT, executed within a distinct region of China. Consequently, extrapolating the findings of this study to more heterogeneous populations or disparate regions may be constrained. It is salient to recognize that our study specifically targeted individuals diagnosed with diabetes, who could have been predisposed to ocular examinations, potentially attenuating the true prevalence of undiagnosed diabetic retinopathy (DR). Moreover, the study’s exclusionary criteria, specifically omitting participants with ungradable retinal images or incomplete questionnaires, introduce a potential selection bias, thereby influencing the study’s outcomes. The reliance on self-reported DR diagnoses further augments the potential for response biases and data inaccuracies. Furthermore, the cross-sectional design of this investigation precludes the establishment of causative relationships, accentuating the exigency for prospective longitudinal studies to corroborate our findings. It is also pivotal to underscore that the analysis did not encompass salient determinants, such as the health insurance status of patients, which could significantly modulate the study’s conclusions.

In conclusion, our study reveals the concerning prevalence of underdiagnosis in cases of DR. Considering the critical significance of early detection, it is imperative to undertake concerted efforts aimed at improving the timely diagnosis of DR. To this end, addressing potential barriers and misconceptions surrounding DR screening and its severity is of paramount importance. Moreover, a key aspect of our approach should involve enhancing patient education, with particular emphasis on older individuals and those with lower educational attainment. By prioritizing these areas, we can actively work towards achieving early detection of DR, thus leading to improved management outcomes.

## Data availability statement

The raw data supporting the conclusions of this article will be made available by the authors, without undue reservation.

## Ethics statement

The studies involving humans were approved by Institutional Review Board of Fushun Eye Hospital. The studies were conducted in accordance with the local legislation and institutional requirements. The participants provided their written informed consent to participate in this study.

## Author contributions

BZ: Conceptualization, Data curation, Investigation, Methodology, Project administration, Resources, Visualization, Writing – original draft. SR: Conceptualization, Investigation, Methodology, Supervision, Writing – review & editing. YW: Funding acquisition, Writing – original draft. DL: Conceptualization, Writing – original draft. XD: Conceptualization, Writing – original draft. DZ: Data curation, Writing – original draft. FW: Writing – review & editing. YL: Writing – review & editing. GZ: Writing – review & editing. KF: Formal analysis, Writing – original draft. ZZ: Writing – original draft.
