# Supplementary material for: Undiagnosed diabetic retinopathy in Northeast China: prevalence and determinants
Source: Front Endocrinol (Lausanne). 2023 Nov 29;14:1263508. doi: 10.3389/fendo.2023.1263508 (PMC10716530; doi:10.3389/fendo.2023.1263508)
Supplement: Supplementary file 1 [file Table_1.docx]

**Supplementary Table 1.** Individual incentives and barriers to diabetic retinopathy screening

| **Item Number** | **Perceptions** |
| --- | --- |
| 1 | People with diabetes are unlikely to get an eye disease. |
| 2 | Diabetes can cause severe eye problems. |
| 3 | There is no treatment for diabetic eye diseases. |
| 4 | I only seek eye care when I am having trouble with my vision. |
| 5 | I receive a reminder from my community health service center when it is time to schedule an eye examination |
| 6 | I think it is important to have an eye examination every year. |
| 7 | I do not want to know if I have an eye disease. |
| 8 | Having an eye examination is not pleasant. |
| 9 | I cannot afford an eye examination. |
| 10 | It is hard for me to travel to an eye doctor. |
| 11 | Lack of a companion for the visit. |
| 12 | Visiting the eye doctor takes too much time. |
| 13 | I have medical problems from diabetes. |
| 14 | my eyes are healthy. |
| 15 | There are many eye doctors where I live. |
| 16 | I want to get an eye examination every year. |
| 17 | People who have good control of their diabetes are unlikely to have eye problems. |
