# Supplementary material for: Undiagnosed diabetic retinopathy in Northeast China: prevalence and determinants
Source: Front Endocrinol (Lausanne). 2023 Nov 29;14:1263508. doi: 10.3389/fendo.2023.1263508 (PMC10716530; doi:10.3389/fendo.2023.1263508)
Supplement: Supplementary file 2 [file Table_2.docx]

**Supplementary Table 2.** Classification of Patients Based on Awareness and Type of Diabetic Retinopathy

| Term |  | Definition |
| --- | --- | --- |
| DR |  | Diagnosis with DR |
| NVTDR |  | Diagnosis with NVTDR |
| VTDR |  | Diagnosis with VTDR |
|  |  |  |
| Undiagnosed DR |  | Diagnosis with DR, unaware of their condition |
| Undiagnosed NVTDR |  | Diagnosis with NVTDR, unaware of their condition |
| Undiagnosed VTDR |  | Diagnosis with VTDR, unaware of their condition |
|  |  |  |
| Diagnosed DR |  | Diagnosis with DR, aware of their condition |
| Diagnosed NVTDR |  | Diagnosis with NVTDR, aware of their condition |
| Diagnosed VTDR |  | Diagnosis with VTDR, aware of their condition |

DR = diabetic retinopathy; NVTDR = non-vision-threatening diabetic retinopathy; VTDR = vision-threatening diabetic retinopathy.
